# Postoperative Telerehabilitation in Patients With Hip Fracture: Systematic Review and Meta-Analysis

**DOI:** 10.2196/77341

**Published:** 2026-03-09

**Authors:** Can-xin Cai, Penny Ping Qin, Chuan-yao Liu, Peng Cai, Xijun Wei

**Affiliations:** 1 Rehabilitation Laboratory of Mixed Reality, Shenzhen Hospital, Southern Medical University, Shenzhen, China; 2 Department of Rehabilitation Medicine, Shenzhen Hospital, Southern Medical University, 13 Xinhu Road, Bao'an District, Shenzhen, 518101, China, 86 755-23360806; 3 Department of Rehabilitation Sciences, Faculty of Health and Social Sciences, Hong Kong Polytechnic University, Hong Kong Special Administrative Region, China (Hong Kong); 4 Department of Occupational Therapy, School of Rehabilitation Sciences, Southern Medical University, Shenzhen, China

**Keywords:** hip fracture, postoperation, telerehabilitation, systematic review, meta-analysis

## Abstract

**Background:**

Telerehabilitation programs use information and communications technologies to facilitate exercise training, self-management education, and health behavior modifications for nonhospitalized patients. In recent years, 2 systematic reviews have examined the effectiveness of telerehabilitation in the recovery of patients with hip fractures but have yielded inconsistent results. This is a significant gap because tools to assess clinical domains such as pain, range of motion, and deformity are crucial for patient outcomes. Moreover, the long-term effects of telerehabilitation on postoperative functional recovery in patients with hip fractures have not been investigated.

**Objective:**

To address the abovementioned research gap, this systematic review aimed to evaluate the short- and long-term effects of telerehabilitation on postoperative functional recovery in patients with hip fractures.

**Methods:**

We searched the PubMed, Cochrane Library, Embase, and Web of Science databases from inception to March 31, 2025. Randomized controlled trials investigating the effect of postoperative telerehabilitation in patients with hip fractures were included in this systematic review. Outcomes of interest included hip function measured using the Harris Hip Score; functional mobility measured using the Short Physical Performance Battery and the timed up and go test; and ability to perform basic activities of daily living measured using the Functional Independence Measure, Barthel index, or modified Barthel index. Meta-analyses were performed using the RevMan software (version 5.3).

**Results:**

A thorough literature search conducted in April 2025 yielded a total of 8 studies involving 740 patients for inclusion in this systematic review. Meta-analysis showed that telerehabilitation was effective for improving the Harris Hip Score at the intervention end point (2 included studies involving 156 participants; mean difference [MD] 7.42, 95% CI 5.61-9.23; *P*<.001; *I*^2^=3%), the Short Physical Performance Battery score at the end point (4 included studies involving 430 participants; MD 1.34, 95% CI 1.14-1.55; *P*<.001; *I*^2^=33%) and at follow-up (2 included studies involving 292 participants; MD 1.17, 95% CI 1.00-1.34; *P*<.001; *I*^2^=0%), the timed up and go score at the end point (4 included studies involving 156 participants; MD –8.45, 95% CI −11.28 to −5.62; *P*<.001; *I*^2^=0%) and at follow-up (2 included studies involving 63 participants; MD −7.66, 95% CI −13.78 to −1.53; *P*=.01; *I*^2^=0%), and ability to perform basic activities of daily living at the end point (5 included studies involving 354 participants; standardized MD 1.65, 95% CI 0.78-2.51; *P*<.001; *I*^2^=91%) and at follow-up (standardized MD 0.43, 95% CI 0.05-0.81; *P*=.03; *I*^2^=48%).

**Conclusions:**

Our review found that postoperative telerehabilitation may benefit short- and long-term functional recovery in patients with hip fractures compared to conventional rehabilitation. However, the evidence was weak due to the limited number and insufficient quality of the included studies and the heterogeneity across the studies.

## Introduction

Hip fractures are a major global public health challenge, with an annual incidence of over 10 million cases [1]. They are most prevalent among adults aged 65 years and older, a population in which fractures are correlated with high mortality rates and a substantial burden on patients and their families [2]. The standard management for hip fractures involves surgical intervention followed by rehabilitation to restore walking ability [3,4]. Despite this, most older patients fail to recover their prefracture level of function, and this persistent impairment may lead to an increased risk of falls [5]. Therefore, long-term postoperative management for older adults with hip fractures is essential to enhance their functional recovery and overall quality of life.

Since the COVID-19 pandemic, telerehabilitation has gained popularity, with physical therapists increasingly willing to incorporate it into their routine practices, prompting health care facilities to adopt innovative methods for engaging with patients. As a branch of telemedicine, telerehabilitation is defined as the use of remote health care methodologies, specifically information and communications technologies, to deliver therapeutic rehabilitation services outside of medical institutions [6]. Telerehabilitation programs use information and communications technologies such as telephones and videoconferencing to facilitate exercise training, self-management, and lifestyle modifications for nonhospitalized patients [7,8].

In recent years, several systematic reviews have been published regarding the effectiveness of telerehabilitation programs for patients with specific chronic diseases, including cardiac, respiratory, and neurological disorders [9,10]. Of note, 2 previous systematic reviews have examined the effectiveness of postoperative telerehabilitation in the recovery of patients with hip fractures, yielding inconsistent results. One review found that, compared to conventional intervention, telerehabilitation was not effective in improving postoperative mobility (as measured using the timed up and go [TUG] test and gait speed) or in alleviating pain but facilitated the ability to perform basic activities of daily living (BADLs) with a clinically irrelevant improvement [11]. Another recently published systematic review found that, compared to traditional rehabilitation, digital health interventions significantly improved postoperative mobility and the ability to perform BADLs, as indicated by the improvement in the TUG performance, the Short Physical Performance Battery (SPPB) score, and the Functional Independence Measure (FIM) score [12]. However, specific measurement tools for hip function, such as the Harris Hip Score (HHS), have not been examined in any systematic review. This is a significant research gap because these tools assess crucial clinical domains, including pain, range of motion, and deformity, that are key outcomes for patients with hip fractures. Moreover, the long-term effects of telerehabilitation on postoperative functional recovery in patients with hip fractures have not been investigated.

Therefore, this systematic review aimed to evaluate the short- and long-term effects of telerehabilitation on postoperative functional recovery in patients with hip fractures. The outcomes of interest included hip function as measured using the HHS [13]; functional mobility as measured using the TUG test [14,15] and SPPB [16-18]; and ability to perform BADLs as measured using the Barthel index (BI), modified BI (MBI), or FIM. We hypothesized that telerehabilitation would be effective for improving postoperative short- and long-term functional recovery in patients with hip fractures compared to conventional rehabilitation.

## Methods

This systematic review and meta-analysis adhered to the PRISMA (Preferred Reporting Items for Systematic Reviews and Meta-Analyses) 2020 guidelines [19], with a registration in PROSPERO (CRD42024498569).

### Literature Search

Two researchers (CC and PPQ) independently searched the PubMed, Embase, Web of Science, and CENTRAL databases from inception to March 31, 2025. The search was limited to English-language literature. Randomized controlled trials investigating the effectiveness of postoperative telerehabilitation treatment for patients with hip fractures were included (Multimedia Appendix 1).

Additional articles were identified by reviewing the reference lists of relevant reviews. The following keywords and the corresponding MeSH (Medical Subject Headings) terms were used: (telerehabilitation OR telerehabilitations OR tele-rehabilitation OR tele-rehabilitations OR remote rehabilitation OR remote rehabilitations OR rehabilitation, remote OR rehabilitations, remote OR virtual rehabilitation OR virtual rehabilitations OR rehabilitation, virtual OR rehabilitations, virtual) AND (hip fractures OR fractures, hip OR intertrochanteric fracture OR intertrochanteric fractures OR trochanteric fractures OR fractures, trochanteric OR femur trochlear fracture OR femur trochlear fractures OR trochlear fracture, femur OR trochlear fractures, femur OR fractures, femur trochlear OR femoral trochlear fracture OR femoral trochlear fractures OR fracture, femoral trochlear OR fractures, femoral trochlear OR trochlear fracture, femoral OR trochlear fractures, femoral OR subtrochanteric fracture OR subtrochanteric fractures).

### Eligibility Criteria

The inclusion criteria were as follows: (1) studies involving participants diagnosed with a hip fracture who were in a stable condition (no further fractures were observed or reported) after surgical intervention; (2) studies involving telerehabilitation as the primary intervention following surgery, including the use of SMS text messaging, telephones, smartphones, and internet-based and software-based methods; (3) studies that reported at least one outcome of interest, including the HHS, TUG test, SPPB score, BI, MBI, and FIM; and (4) randomized controlled trials.

The exclusion criteria were (1) only study protocol or conference abstract available and (2) unavailability of the full text.

### Study Selection

The search results were imported into EndNote X9 (Clarivate Analytics) for duplicate removal. To ensure the accuracy of this process, 2 authors (CC and PPQ) manually verified the deleted duplicates. The titles and abstracts of the remaining records were then screened independently by these authors (CC and PPQ). Full texts were retrieved for further review if deemed eligible by either author. Any disagreements between the reviewers were resolved through discussion with a third author (CL).

### Data Extraction

Two authors (CC and PPQ) independently extracted data from the included studies. The extracted data encompassed the following: basic study information (the first author, publication year, and country), participant baseline characteristics (sample size, age, and gender), details of the telerehabilitation and control interventions, and outcome measures (including follow-up period and measurement tools).

### Quality Evaluation

Two authors (CC and PPQ) independently evaluated the quality of the included studies using version 2 of the Cochrane risk-of-bias tool for randomized trials [20]. The risk-of-bias assessment consisted of the following domains: (1) randomization process, (2) deviations from intended interventions, (3) missing outcome data, (4) measurement of the outcome, and (5) selection of the reported results. The overall risk of bias was classified as low risk of bias, some concerns, and high risk of bias.

### Data Analysis

The Cochrane Collaboration’s Review Manager software (version 5.3) was used for all statistical analyses. The overall estimate of the treatment effect was calculated using the means and SDs of outcome scores for continuous data in the telerehabilitation intervention and control groups. Short- and long-term effects were analyzed by comparing the differences in outcome scores between the 2 groups at the intervention end point and at the last follow-up time point, respectively. For studies using the same measurement tool, we calculated a pooled estimate of the mean differences (MDs) with 95% CIs. In cases in which different measurement tools were used, we used standardized MDs (SMDs).

To investigate potential clinical and methodological heterogeneity across the included studies, the following factors were summarized and compared: (1) patient characteristics (age and baseline scores of the outcomes of interest), (2) characteristics of telerehabilitation (duration, frequency, and delivery mode), (3) type of control intervention, (4) follow-up period for the outcomes of interest, and (5) risk of bias. Where low clinical and methodological heterogeneity was found across the studies in a given meta-analysis, a fixed-effects model was used; otherwise, a random-effects model was applied. Statistical heterogeneity was quantified using the *I*^2^ statistic. *I*^2^ values greater than 50% were considered to indicate substantial heterogeneity [21]. When substantial statistical heterogeneity was present and a sufficient number of studies were available, subgroup analyses were performed based on the abovementioned factors contributing to clinical or methodological heterogeneity. Furthermore, a sensitivity analysis was planned to assess the impact on the pooled results provided that a sufficient number of studies were available. This was performed by excluding studies with a high risk of bias or those that differed substantially from others in the factors considered for subgroup analyses.

## Results

### Study Identification

**Figure 1.**
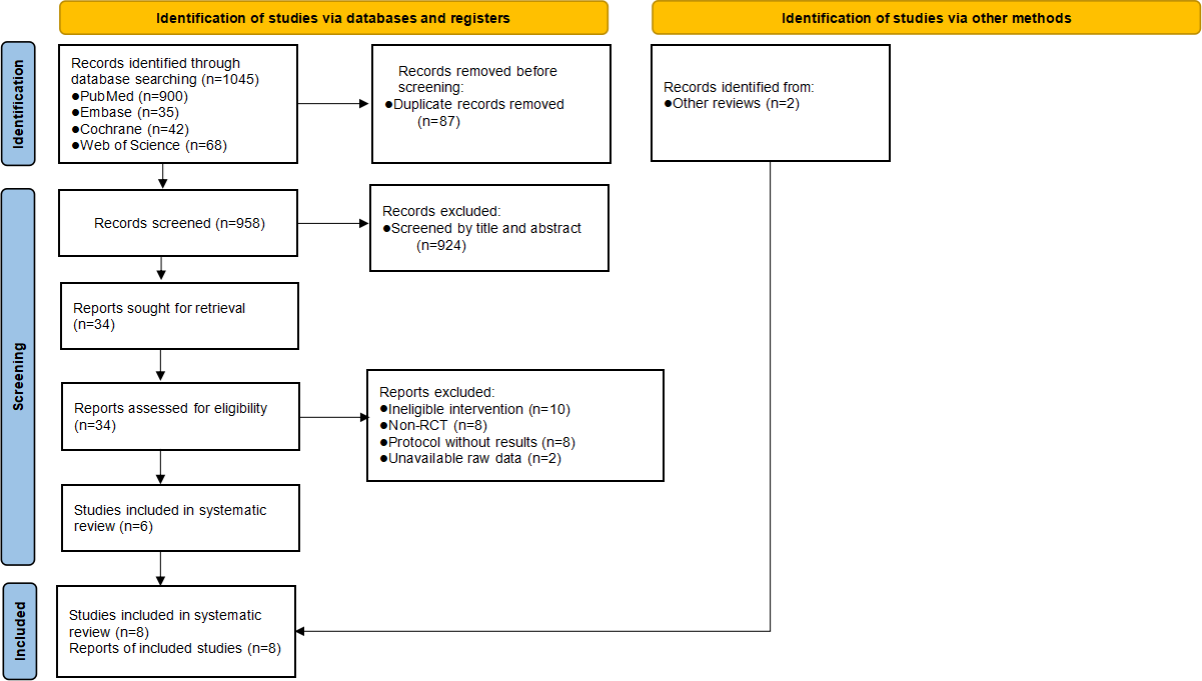
Flow diagram for the systematic search. RCT: randomized controlled trial.

The initial database search yielded a total of 1045 records, of which 6 (0.6%) met the eligibility criteria and were included. A further 2 studies were identified by checking the reference lists of relevant reviews [11], resulting in a total of 8 studies for the systematic review and meta-analysis. The study selection process is illustrated in the PRISMA flow diagram (Figure 1).

### Study Characteristics

The 8 included studies comprised 740 patients, including 569 female and 161 male individuals. The sample sizes varied from 31 to 232 in the individual studies.

The treatment strategies for postoperative telerehabilitation for patients with hip fractures included strengthening, balance, coordination, stretching exercises, BADL training, introduction of walking aids, and safety education. Most included studies provided telerehabilitation through mobile apps or software [5,22-28] supervised by professionals remotely, one study used a DVD supervised via telephone calls [27], and one study used a telephone call as the primary strategy [28]. The control interventions comprised home-based exercise through written materials [5,25,26], in-person rehabilitation [23,24], follow-up assessment through telephone [22] and health nutrition education [27], and no intervention [28]. The duration of telerehabilitation varied from 35 minutes to 6 months. The follow-up periods varied from 3 weeks to 9 months [23,25-28]. Table 1 presents the main characteristics of the included studies [5,22-28].

**Table 1.** Characteristics of the included studies.

Study and country	Participants	Telerehabilitation	Control intervention	Outcome measures	Time points of outcome measurement
Kalron et al [26], 2018, Israel	Sample size: 15 in telerehabilitation vs 17 in control group (female: 10/15 vs 9/17, respectively); age: mean 65.7, SD 7.8 y vs mean 67.3, SD 9.5 y	Home-based training program through software, including exercises for movement, strengthening the lower limbs, and balance; 40-50 min per session, 3 times per week for 6 weeks Supervised by professionals through the software	Home-based exercises through an exercise booklet (exercises were similar to those in the telerehabilitation group)	TUG^a^ test	Intervention end point: 6 weeks; follow-up: 4 weeks after the intervention
Li et al [25], 2022, China	Sample size: 15 in telerehabilitation vs 16 in control group (female: 14/15 vs 11/16, respectively); age: mean 76.5, SD 8.6 y vs mean 82.1, SD 9.7 y	Home-based training program through mobile app, including trunk and lower-extremity strengthening and stretching, coordination, balance, and functional exercises related to ADLs^b^, for 3 weeks Supervised by professionals through the mobile app	Home-based training through written sheets (exercises were equivalent to those in the telerehabilitation group)	TUG test and MBI^c^	Intervention end point: 3 weeks; follow-up: 3 weeks after the intervention
Ortiz-Piña et al [24], 2021, Spain	Sample size: 35 in telerehabilitation vs 36 in control group (female: 26/35 vs 27/36, respectively); age: mean 76.71, SD 6.04 y vs mean 80.72, SD 5.59 y	Home-based training through software, including lower- and upper-body strengthening, balance, cardiovascular exercises, and occupational therapy (ADL training and introduction to walking aids); 50-60 min per session for 12 weeks Supervised by caregivers	Home-based in-person rehabilitation, including 5-15 sessions of physiotherapy and occupational therapy	TUG test, SPPB^d^, and FIM^e^	Intervention end point: 12 weeks; follow-up: N/A^f^
Wu et al [5], 2022, China	Sample size: 43 in telerehabilitation vs 42 in control group (female: 30/43 vs 31/42, respectively); age: mean 74.28, SD 5.06 y vs mean 72.0, SD 6.77 y	Home-based training through software, including cardiopulmonary function exercise and balance exercises, ADL training, and introduction to walking aids, for 6 months Supervised by professionals through the software and communication with professionals through videoconferencing	Home-based rehabilitation through a written text and communication with professionals through phone calls and outpatient service	HHS^g^ and FIM	Intervention end point: 6 months; follow-up: N/A
Zhang et al [22], 2022, China	Hip replacement group sample size: 17 in telerehabilitation vs 14 in control group; internal fixation group sample size: 10 in telerehabilitation vs 10 in control group	Home-based training through a mobile app, including education and walking, balance, and stair-walking exercises, for 3 months Supervised by professionals through the mobile app	Follow-up assessment through telephone	HHS, FIM, TUG test, and SPPB	During the intervention period: at 2 weeks and 2 months; intervention end point: 3 months; follow-up: N/A
Prieto-Moreno et al [23], 2024, Portugal	Sample size: 51 in telerehabilitation vs 54 in control group (female: 37/51 vs 38/54, respectively); age: mean 79.55, SD 7.11 y vs mean 80.07, SD 7.74 y	Home-based training through software, including lower- and upper-body strengthening, balance, cardiovascular exercises, and occupational therapy (ADL training and introduction to walking aids); 3 sessions per week for 12 weeks Supervised by professionals through the software	Home-based in-person rehabilitation, including 5-15 sessions of physiotherapy and occupational therapy	SPPB and FIM	Intervention end point: 3 months; follow-up: 9 months after the intervention
Latham et al [27], 2014, United States	Sample size: 120 in telerehabilitation vs 112 in control group (female: 83/120 vs 77/112, respectively); age: mean 77.2, SD 10.2 y vs mean 78.9, SD 9.4 y	Home-based training through DVD, including strengthening and standing exercises; 3 times per week for 6 months Supervised by professionals through telephone	Nutrition education for cardiovascular health through telephone	SPPB	Intervention end point: 6 months; follow-up: 3 months after the intervention
Di Monaco et al [28], 2015, Italy	Sample size: 78 in telerehabilitation vs 75 in control group (female: 78/78 vs 75/75, respectively); age: mean 78.7, SD 7.2 y vs mean 79.3, SD 8.0 y	A telephone call to check environmental hazards, behaviors in ADLs, and use of assistive devices and reinforced targeted modifications to prevent falls for 35 minutes	No intervention	BI^h^	Intervention end point: N/A; follow-up: 5.5 months after the intervention

a TUG: timed up and go.

b ADL: activity of daily living.

c MBI: modified Barthel index.

d SPPB: Short Physical Performance Battery.

e FIM: Functional Independence Measure.

f N/A: not applicable.

g HHS: Harris Hip Score.

h BI: Barthel index.

### Risk of Bias in the Included Studies

**Figure 2.**
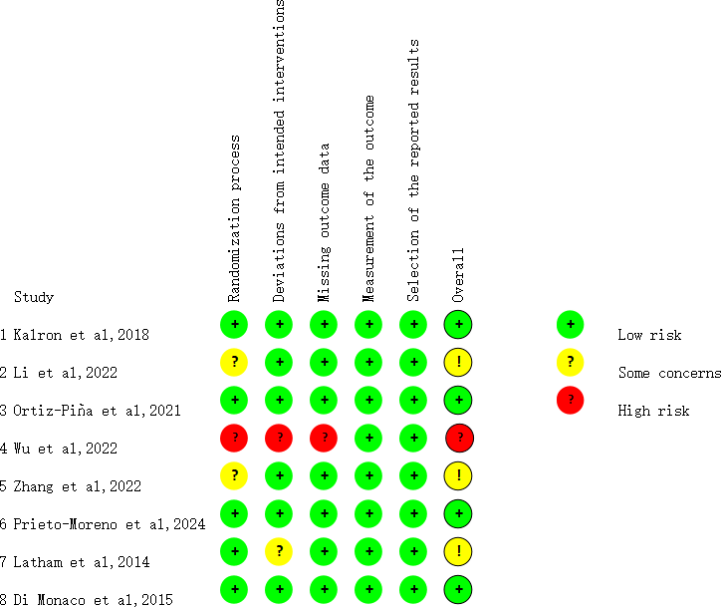
A detailed breakdown of the methodological quality assessment for the included studies. Overall, 4 studies exhibited a low risk of bias, 3 raised some concerns, and 1 exhibited a high risk of bias [5,22-28].

Four studies [23,24,26,28] had a low risk of bias, 3 [22,25,27] had some concerns, and 1 [5] had a high risk of bias. Overall, the randomization process was the most problematic domain as 3 studies poorly described the method used [5,22,25]. The results of the methodological quality assessment are provided in Figure 2 [5,22-28].

### Effectiveness of Postoperative Telerehabilitation on Functional Recovery at the Intervention End Point and Follow-Up

**Figure 3.**
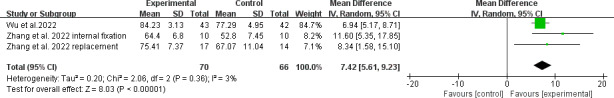
Forest plot of Harris Hip Score (HHS) results at the intervention end point. The analysis yielded a positive result with a mean difference of 7.42 (95% CI 5.61-9.23; *P*<.001; *I*^2^=3%) [5,22]. IV: inverse variance.

HHS was measured in 2 studies involving a total of 156 participants at the intervention end point [5,22]. Low clinical heterogeneity was observed in patient characteristics (mean age range 72-77 years; comparable baseline mean HHS between 34.9 and 43.6). However, substantial clinical heterogeneity was found regarding the duration of telerehabilitation (6 months [5] vs 3 months [22]). A random-effects meta-analysis yielded a statistically significant, positive result (MD 7.42, 95% CI 5.61-9.23; *P*<.001; Figure 3 [5,22]) with low statistical heterogeneity (*I*^2^=3%; *P*=.36; Figure 3 [5,22]).

**Figure 4.**
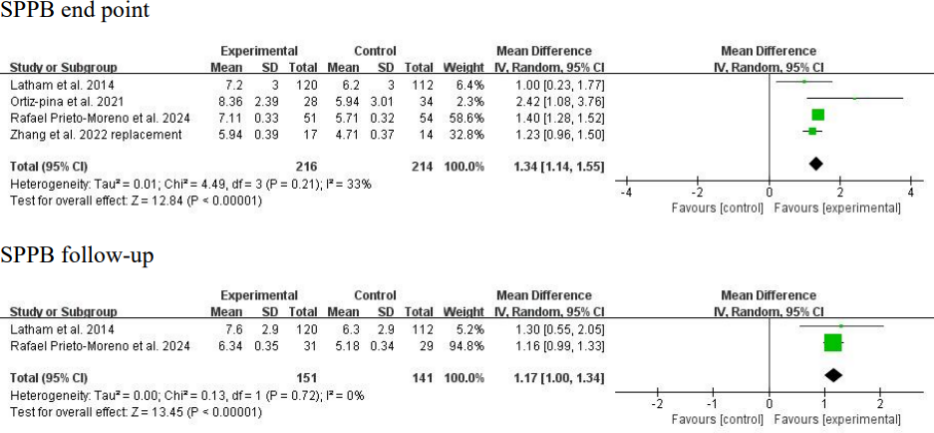
Forest plot of Short Physical Performance Battery (SPPB) results at the intervention end point and follow-up. The analysis yielded a positive result at the intervention end point with a mean difference of 1.34 (95% CI 1.14-1.55; *P*<.001; *I*^2^=33%). The analysis yielded a positive result at follow-up with a mean difference of 1.17 (95% CI 1.00-1.34; *P*<.001; *I*^2^=0%) [22-24,27]. IV: inverse variance.

SPPB was measured in 4 studies (n=430 participants) at the intervention end point [22-24,27]. Considerable clinical heterogeneity was observed in baseline SPPB scores (mean scores 4.71-5.9 [22,27] vs 2.69-3.21 [23,24]). Furthermore, both the duration of telerehabilitation (3 months [22-24] vs 6 months [27]) and the type of control intervention (telephone assessment [22,27] vs in-person rehabilitation [23,24]) varied across studies. A random-effects meta-analysis showed a significant positive result at the intervention end point (MD 1.34, 95% CI 1.14-1.55; *P*<.001; Figure 4 [22-24,27]) with low to moderate statistical heterogeneity (*I*^2^=33%; *P*=.21; Figure 4 [22-24,27]). SPPB was measured in 2 studies (n=292 participants) at follow-up [23,27]. In addition to the aforementioned clinical heterogeneity, great methodological heterogeneity was noted in the follow-up period (3 months [27] vs 9 months after the intervention [23]). The random-effects analysis remained significant at follow-up (MD 1.17, 95% CI 1.00-1.34; *P*<.001; Figure 4 [22-24,27]) with low statistical heterogeneity (*I*^2^=0%; *P*=.72; Figure 4 [22-24,27]).

**Figure 5.**
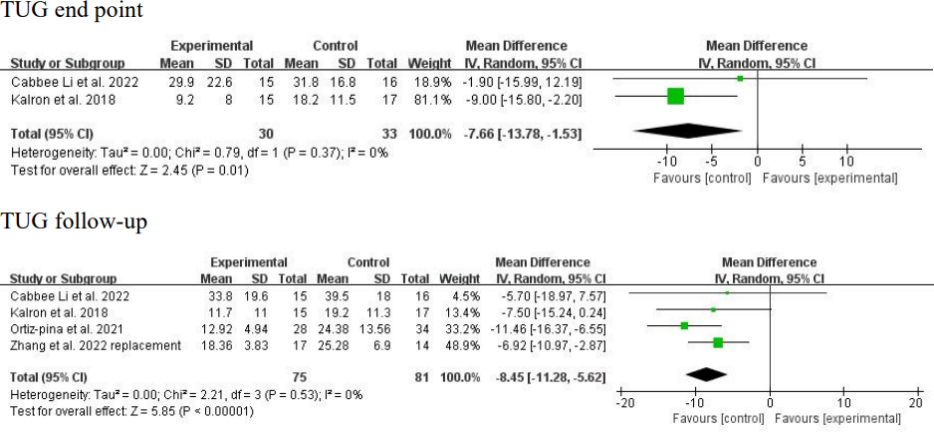
Forest plot of timed up and go (TUG) test results at the intervention end point and follow-up. The analysis yielded a positive result at the intervention end point with a mean difference of –8.45 (95% CI –11.28 to –5.62; *P*<.001; *I*^2^=0%). The analysis yielded a positive result at follow-up with a mean difference of –7.66 (95% CI –13.78 to –1.53; *P*=.01; *I*^2^=0%) [22,24-26]. IV: inverse variance.

The TUG test was measured in 4 studies (n=156 participants) at the intervention end point [22,24-26]. Considerable clinical heterogeneity was found regarding the patient characteristics (mean age 65.7-67.3 years [26] vs 75.17-82.1 years [22,24,25]; mean baseline TUG score 21.4-25.6 [26] vs 39.7-45.2 [25] vs 66.53-99.72 [24]; data unavailable for the study by Zhang et al [22]). The duration of telerehabilitation (3-6 weeks [25,26] vs 3 months [22,24]) and type of control intervention (written materials [25,26] vs telephone assessment [22] vs in-person rehabilitation [24]) also varied. A random-effects meta-analysis demonstrated a significant positive result at the intervention end point (MD –8.45, 95% CI −11.28 to −5.62; *P*<.001; Figure 5) with low statistical heterogeneity (*I*^2^=0%; *P*=.53; Figure 5 [22,24-26]). The TUG test was measured in 2 studies (n=63 participants) at follow-up [25,26]. Low methodological heterogeneity was observed in the follow-up period (3-4 weeks after the intervention [25,26]) and risk of bias (low to moderate risk of bias [25,26]). However, due to the considerable clinical heterogeneity noted above (in patient age and baseline function), a random-effects model was applied. This analysis yielded a significant positive result at follow-up (MD −7.66, 95% CI −13.78 to −1.53; *P*=.001; *I*^2^=0%; *P*=.37; Figure 5 [22,24-26]).

**Figure 6.**
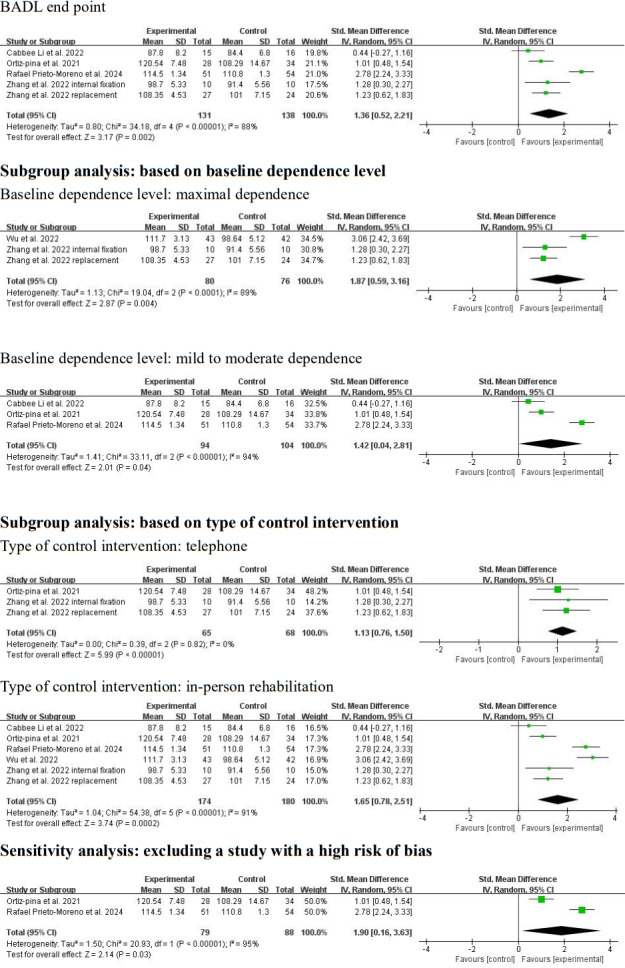
Forest plot of basic activity of daily living (BADL) results at the intervention end point. The analysis yielded a positive result at the intervention end point with a standardized mean difference of 1.65 (95% CI 0.78-2.51; *P*<.001) and high heterogeneity (*I*^2^=91%). Subgroup analyses were conducted based on baseline dependence level and type of control intervention. A sensitivity analysis was conducted by excluding a study with a high risk of bias [5,22-25]. IV: inverse variance.

**Figure 7.**
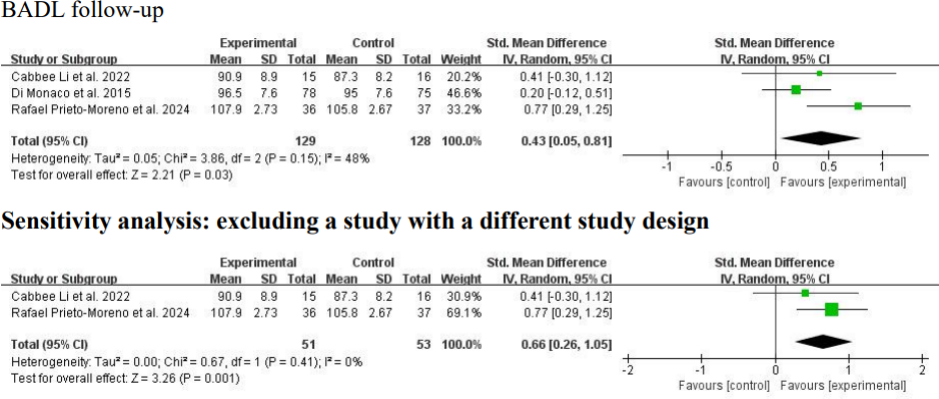
Forest plot of basic activity of daily living (BADL) results at follow-up. The analysis yielded a positive result at follow-up with a standardized mean difference of 0.43 (95% CI 0.05-0.81; *P*=.03; *I*^2^=48%). A sensitivity analysis was conducted by excluding a study with a different study design from the others [23,25,28]. IV: inverse variance.

Because various BADL measurement tools were used across the included studies, SMDs were used as the effect size measure. Zhang et al [22] reported BADL outcomes separately for the hip replacement and internal fixation groups. BADLs were measured in 5 studies (n=354 participants) at the intervention end point [5,22-25]. Considerable clinical heterogeneity was observed regarding patient characteristics (baseline dependence level: maximal dependence [5,22] vs mild to moderate dependence [23-25]), duration of telerehabilitation (3 weeks [25] vs 3 months [22-24] vs 6 months [5]), and type of control intervention (written materials [25] vs telephone assessment [5,22] vs in-person rehabilitation [23,24]). In addition, substantial methodological heterogeneity was observed in the risk of bias (high [5] vs some concerns [22,25] vs low [23,24]). A random-effects meta-analysis yielded a significant positive result with high heterogeneity (SMD 1.65, 95% CI 0.78-2.51; *P*<.001; *I*^2^=91%; *P*<.001; Figure 6 [5,22-25]). Subgroup analyses were conducted based on baseline dependence level, duration of telerehabilitation, and type of control intervention. For studies involving patients with a high baseline dependence level [5,22], the analysis showed a positive result with high heterogeneity (SMD 1.87, 95% CI 0.59-3.16; *P*=.004; *I*^2^=89%; *P*<.001; Figure 6 [5,22-25]). For studies with a lower baseline dependence level [23-25], the result was also positive with high heterogeneity (SMD 1.42, 95% CI 0.04-2.81; *P*=.04; *I*^2^=94%; *P*<.001; Figure 6 [5,22-25]). Subgroup analysis by duration of telerehabilitation was not possible due to an insufficient number of studies. For studies with telephone-based assessment as a control intervention [5,22], the result was positive with high heterogeneity (SMD 1.87, 95% CI 0.59-3.16; *P*=.004; *I*^2^=89%; *P*<.001; Figure 6 [5,22-25]). For studies with in-person rehabilitation as the control [23,24], the result was similarly positive with high heterogeneity (SMD 1.90, 95% CI 0.16-3.63; *P*=.03; *I*^2^=95%; *P*<.001; Figure 6 [5,22-25]). A sensitivity analysis was conducted by (1) excluding 2 studies with intervention durations shorter [25] or longer [5] than 3 months and (2) excluding 1 study with a high risk of bias [5]. Pooling the 3 remaining studies with a 3-month intervention duration [22-24] using a random-effects model yielded a positive result with low statistical heterogeneity (SMD 1.13, 95% CI 0.76-1.50; *P*<.001; *I*^2^=0%; *P*=.82; Figure 6 [5,22-25]). Pooling the 4 studies with low to moderate risk of bias [22-25] yielded a positive result with high heterogeneity (SMD 1.36, 95% CI 0.52-2.21; *P*=.002; *I*^2^=88%; *P*<.001). BADLs were measured in 3 studies (n=257 participants) at follow-up [23,25,28]. Considerable clinical heterogeneity was observed regarding baseline dependence level (mild to moderate dependence [23,25] vs minimal dependence [28]), duration of telerehabilitation (2.5-3 weeks [25,28] vs 12 weeks [23]), delivery mode of telerehabilitation (telephone based [28] vs software based [23,25]), and type of control intervention (no intervention [28] vs in-person rehabilitation [23] vs written materials [25]). Moreover, substantial methodological heterogeneity was found in the follow-up period (3 weeks [25] vs 5.5 months [28] vs 9 months [23] after the intervention). A random-effects model yielded a positive result with moderate heterogeneity at follow-up (SMD 0.43, 95% CI 0.05-0.81; *P*=.03; *I*^2^=48%; *P*=.15; Figure 7 [23,25,28]). A sensitivity analysis excluded 1 study [28] that differed substantially from the others in design (baseline dependence level, type of telerehabilitation, and type of control intervention). A random-effects model for the remaining 2 studies [23,25] yielded a positive result with low heterogeneity (SMD 0.66, 95% CI 0.26-1.05; *P*=.001; *I*^2^=0%; *P*=.41; Figure 7 [23,25,28]).

## Discussion

### Principal Findings

Our review found that postoperative telerehabilitation was effective in improving short- and long-term hip function, functional mobility, and ability to perform BADLs in patients with hip fractures compared to conventional rehabilitation. However, current evidence was weak due to the limited number and insufficient quality of the included studies and the heterogeneity across studies.

Our findings align with previous evidence that telerehabilitation is beneficial to short-term recovery of functional mobility and ability to perform BADLs at posttreatment compared to usual care [12]. Moreover, our review found that hip function was also improved after telerehabilitation. In addition, our findings revealed the effect of telerehabilitation on long-term recovery (3 weeks to 9 months after the intervention) in functional mobility and the ability to perform BADLs. The effectiveness in improving BADLs showed substantial heterogeneity across the included studies in the postintervention analysis. Although subgroup analyses were conducted based on the type of control intervention and the baseline dependence level, they failed to reduce the heterogeneity. A potential source of heterogeneity may be the duration of telerehabilitation as the statistical heterogeneity decreased dramatically when 2 studies with telerehabilitation shorter (3 weeks) [25] and longer (6 months) [5] than 3 months were excluded. Another potential source of heterogeneity may be differences in adherence to the telerehabilitation programs. Although only 2 of the 5 studies in the BADL analysis reported adherence rates for telerehabilitation, the substantial difference (87% [25] vs 15% [24]) suggests considerable variability in compliance across studies.

This systematic review revealed that telerehabilitation strategies, including exercises for strengthening the lower extremities, balance, coordination, stretching, and BADL training, introduction of walking aids, and safety education are commonly used for patients with hip fractures. According to clinical practice guidelines, postoperative progressive resistance exercises and balance training are strongly recommended, and BADL training and complication prevention are suggested for older patients with hip fractures [29]. However, it is essential to standardize these training programs to mitigate the risk of adverse events [30,31].

Regarding the delivery mode of telerehabilitation, most studies used software- or mobile app–based telerehabilitation with remote supervision by professionals, and only 2 studies conducted before 2015 used DVD-based or telephone-based telerehabilitation. Recently, the application of virtual reality has become an increasingly common approach in telerehabilitation, with superior long-term benefits for patients with total knee replacements compared with traditional rehabilitation [32]. Although all these remote rehabilitation approaches could facilitate adherence to postoperative management, reduce costs, and decrease the burden on therapists and caregivers, the efficacy of different delivery strategies could vary. Future studies are needed to compare the cost-effectiveness ratio between different delivery modes of telerehabilitation.

Our findings may have several clinical implications. First, substantial clinically important improvements in functional mobility, as measured using the SPPS [33] and TUG test [34,35], were observed following telerehabilitation compared to usual care at the postintervention and follow-up time points. However, the overall improvement in HHS (7.35 points) fell below the minimal clinically important improvement threshold (15.9-18 points) [36]. For BADLs, a large short-term effect size (SMD 1.65) was found, but this decreased to a small to moderate effect at the long-term follow-up (SMD 0.43). Furthermore, no included studies demonstrated a clinically relevant improvement for BADLs (21 points on the FIM [37] and 9.8 points on the BI [38]). These findings indicate that telerehabilitation may be more beneficial for functional mobility than for hip function or BADLs. Second, we noted that most included studies were conducted in countries with established research environments (Israel, Spain, Portugal, and the United States), with China being the only included country with an emerging research landscape. Therefore, the generalizability of telerehabilitation requires further investigation, specifically in terms of its feasibility and effectiveness in low- and middle-income countries. Third, the included studies exhibited variable follow-up periods (3 weeks to 9 months), which impedes the investigation of long-term effects. Future studies could adopt a more standardized follow-up schedule (eg, every 3 months after the intervention). Fourth, adherence to telerehabilitation was poorly reported in the included studies, leaving its influence on the effect size unclear. As a key proposed benefit of telerehabilitation is the facilitation of prolonged rehabilitation training—and studies have demonstrated higher compliance in telerehabilitation groups, potentially leading to greater improvement [22,26]—detailed adherence data are crucial for interpreting effectiveness. Future studies should report adherence and compliance rates in detail and develop effective supervision approaches to ensure the efficacy of telerehabilitation.

### Limitations

This study has several limitations. First, it only included literature published in English, which may have omitted relevant evidence from articles published in other languages, potentially introducing language bias and limiting the generalizability of the findings. Second, the small number of included studies and the substantial heterogeneity observed influenced the strength of the evidence. Third, no statistical analysis for publication bias was conducted due to the small number of included studies.

### Conclusions

Telerehabilitation is effective for short- and long-term functional recovery in postoperative patients with hip fractures compared to conventional rehabilitation. However, the evidence is weak due to the limited number of included studies and the high heterogeneity across studies. Future high-quality studies with larger sample sizes are needed to investigate the effectiveness of telerehabilitation. These studies should report the intervention strategies, delivery modes, and adherence rates of interventions in detail; incorporate comprehensive outcome measures for hip function (eg, HHS), functional mobility (eg, SPPS and TUG test), and ability to perform BADLs (eg, BI, MBI, and FIM); and apply a relatively standard follow-up period to investigate the long-term effect of telerehabilitation.

## Supplementary material

10.2196/77341 Multimedia Appendix 1 Search strategy for PubMed.

10.2196/77341 Checklist 1 PRISMA checklist.

## Abbreviations

BADL: basic activity of daily living
BI: Barthel index
FIM: Functional Independence Measure
HHS: Harris Hip Score
MBI: modified Barthel index
MD: mean difference
MeSH: Medical Subject Headings
PRISMA: Preferred Reporting Items for Systematic Reviews and Meta-Analyses
SMD: standardized mean difference
SPPB: Short Physical Performance Battery
TUG: timed up and go

## References

[1] Sing CW, Lin TC, Bartholomew S (2023). Global epidemiology of hip fractures: secular trends in incidence rate, post-fracture treatment, and all-cause mortality. J Bone Miner Res.

[2] Tebé C, Martínez-Laguna D, Carbonell-Abella C (2019). The association between type 2 diabetes mellitus, hip fracture, and post-hip fracture mortality: a multi-state cohort analysis. Osteoporos Int.

[3] Handoll HH, Sherrington C, Mak JC (2011). Interventions for improving mobility after hip fracture surgery in adults. Cochrane Database Syst Rev.

[4] Handoll HH, Cameron ID, Mak JC, Panagoda CE, Finnegan TP (2021). Multidisciplinary rehabilitation for older people with hip fractures. Cochrane Database Syst Rev.

[5] Wu WY, Zhang YG, Zhang YY, Peng B, Xu WG (2023). Clinical effectiveness of home-based telerehabilitation program for geriatric hip fracture following total hip replacement. Orthop Surg.

[6] Duruturk N (2020). Telerehabilitation intervention for type 2 diabetes. World J Diabetes.

[7] Peretti A, Amenta F, Tayebati SK, Nittari G, Mahdi SS (2017). Telerehabilitation: review of the state-of-the-art and areas of application. JMIR Rehabil Assist Technol.

[8] Wong AKC, Wong FKY, Chow KKS, Wong SM, Lee PH (2021). Effect of a telecare case management program for older adults who are homebound during the COVID-19 pandemic: a pilot randomized clinical trial. JAMA Netw Open.

[9] Lee AYL, Wong AKC, Hung TTM, Yan J, Yang S (2022). Nurse-led telehealth intervention for rehabilitation (telerehabilitation) among community-dwelling patients with chronic diseases: systematic review and meta-analysis. J Med Internet Res.

[10] Truijen S, Abdullahi A, Bijsterbosch D (2022). Effect of home-based virtual reality training and telerehabilitation on balance in individuals with Parkinson disease, multiple sclerosis, and stroke: a systematic review and meta-analysis. Neurol Sci.

[11] Tsuge T, Yamamoto N, Taito S, Miura T, Shiratsuchi D, Yorifuji T (2025). Efficacy of telerehabilitation for patients after hip fracture surgery: a systematic review and meta-analysis. J Telemed Telecare.

[12] Pliannuom S, Pinyopornpanish K, Buawangpong N (2024). Characteristics and effects of home-based digital health interventions on functional outcomes in older patients with hip fractures after surgery: systematic review and meta-analysis. J Med Internet Res.

[13] Harris WH (1969). Traumatic arthritis of the hip after dislocation and acetabular fractures: treatment by mold arthroplasty. An end-result study using a new method of result evaluation. J Bone Joint Surg Am.

[14] Podsiadlo D, Richardson S (1991). The timed “Up & Go”: a test of basic functional mobility for frail elderly persons. J Am Geriatr Soc.

[15] Bohannon RW (2006). Reference values for the timed up and go test: a descriptive meta-analysis. J Geriatr Phys Ther.

[16] Volpato S, Cavalieri M, Sioulis F (2011). Predictive value of the short physical performance battery following hospitalization in older patients. J Gerontol A Biol Sci Med Sci.

[17] Soubra R, Chkeir A, Novella JL (2019). A systematic review of thirty-one assessment tests to evaluate mobility in older adults. Biomed Res Int.

[18] Pavasini R, Guralnik J, Brown JC (2016). Short physical performance battery and all-cause mortality: systematic review and meta-analysis. BMC Med.

[19] Page MJ, McKenzie JE, Bossuyt PM (2021). The PRISMA 2020 statement: an updated guideline for reporting systematic reviews. Syst Rev.

[20] Sterne JAC, Savović J, Page MJ (2019). RoB 2: a revised tool for assessing risk of bias in randomised trials. BMJ.

[21] Higgins JP, Thomas J, Chandler J (2024). Cochrane Handbook for Systematic Reviews of Interventions Version 65.

[22] Zhang YY, Zhang YG, Li Z, Li SH, Xu WG (2022). Effect of home-based telerehabilitation on the postoperative rehabilitation outcome of hip fracture in the aging population. Orthop Surg.

[23] Prieto-Moreno R, Mora-Traverso M, Estévez-López F (2024). Effects of the ActiveHip+ mHealth intervention on the recovery of older adults with hip fracture and their family caregivers: a multicentre open-label randomised controlled trial. EClinicalMedicine.

[24] Ortiz-Piña M, Molina-Garcia P, Femia P (2021). Effects of tele-rehabilitation compared with home-based in-person rehabilitation for older adult’s function after hip fracture. Int J Environ Res Public Health.

[25] Li CT, Hung GK, Fong KN, Gonzalez PC, Wah SH, Tsang HW (2022). Effects of home-based occupational therapy telerehabilitation via smartphone for outpatients after hip fracture surgery: a feasibility randomised controlled study. J Telemed Telecare.

[26] Kalron A, Tawil H, Peleg-Shani S, Vatine JJ (2018). Effect of telerehabilitation on mobility in people after hip surgery: a pilot feasibility study. Int J Rehabil Res.

[27] Latham NK, Harris BA, Bean JF (2014). Effect of a home-based exercise program on functional recovery following rehabilitation after hip fracture. JAMA.

[28] Di Monaco M, De Toma E, Gardin L, Giordano S, Castiglioni C, Vallero F (2015). A single postdischarge telephone call by an occupational therapist does not reduce the risk of falling in women after hip fracture: a randomized controlled trial. Eur J Phys Rehabil Med.

[29] Min K, Beom J, Kim BR (2021). Clinical practice guideline for postoperative rehabilitation in older patients with hip fractures. Ann Rehabil Med.

[30] Rosner BI, Gottlieb M, Anderson WN (2018). Effectiveness of an automated digital remote guidance and telemonitoring platform on costs, readmissions, and complications after hip and knee arthroplasties. J Arthroplasty.

[31] Nelson M, Russell T, Crossley K, Bourke M, McPhail S (2021). Cost-effectiveness of telerehabilitation versus traditional care after total hip replacement: a trial-based economic evaluation. J Telemed Telecare.

[32] Gazendam A, Zhu M, Chang Y, Phillips S, Bhandari M (2022). Virtual reality rehabilitation following total knee arthroplasty: a systematic review and meta-analysis of randomized controlled trials. Knee Surg Sports Traumatol Arthrosc.

[33] Perera S, Mody SH, Woodman RC, Studenski SA (2006). Meaningful change and responsiveness in common physical performance measures in older adults. J Am Geriatr Soc.

[34] Wright AA, Cook CE, Baxter GD, Dockerty JD, Abbott JH (2011). A comparison of 3 methodological approaches to defining major clinically important improvement of 4 performance measures in patients with hip osteoarthritis. J Orthop Sports Phys Ther.

[35] Yuksel E, Unver B, Kalkan S, Karatosun V (2021). Reliability and minimal detectable change of the 2-minute walk test and timed up and go test in patients with total hip arthroplasty. Hip Int.

[36] Singh JA, Schleck C, Harmsen S, Lewallen D (2016). Clinically important improvement thresholds for Harris Hip Score and its ability to predict revision risk after primary total hip arthroplasty. BMC Musculoskelet Disord.

[37] Arcolin I, Godi M, Giardini M, Guglielmetti S, Bellotti L, Corna S (2024). Minimal clinically important difference of the functional independence measure in older adults with hip fracture. Disabil Rehabil.

[38] Unnanuntana A, Jarusriwanna A, Nepal S (2018). Validity and responsiveness of Barthel index for measuring functional recovery after hemiarthroplasty for femoral neck fracture. Arch Orthop Trauma Surg.

