# Supplementary material for: Postoperative Telerehabilitation in Patients With Hip Fracture: Systematic Review and Meta-Analysis
Source: JMIR Mhealth Uhealth. 2026 Mar 9;14:e77341. doi: 10.2196/77341 (PMC12978912; doi:10.2196/77341)
Supplement: Multimedia Appendix 1 [file mhealth-v14-e77341-s001.docx]

**Appendix 1. Search strategy of PubMed through PubMed**

| #1 | "Telerehabilitation" |
| --- | --- |
| #2 | "Telerehabilitations" |
| #3 | "Tele-rehabilitation" |
| #4 | "Tele rehabilitation" |
| #5 | "Tele-rehabilitations" |
| #6 | "Remote Rehabilitation" |
| #7 | "Rehabilitation, Remote" |
| #8 | "Rehabilitations, Remote" |
| #9 | "Remote Rehabilitations" |
| #10 | "Virtual Rehabilitation" |
| #11 | "Rehabilitation, Virtual" |
| #12 | "Rehabilitations, Virtual" |
| #13 | "Virtual Rehabilitations" |
| #14 | #1 OR #2 OR #3 OR #4 OR #5 OR #6 OR #7 OR #8 OR #9 OR #10 OR #11 OR #12 OR #13 |
| #15 | "Hip Fractures" |
| #16 | "Fractures, Hip" |
| #17 | "Intertrochanteric Fractures" |
| #18 | "Intertrochanteric Fractures" |
| #19 | "Trochanteric Fractures" |
| #20 | "Fractures, Trochanteric" |
| #21 | "Trochlear Fractures, Femur" |
| #22 | "Femur Trochlear Fracture" |
| #23 | "Femur Trochlear Fractures" |
| #24 | "Femur Trochlear Fractures" |
| #25 | "Fractures, Femur Trochlear" |
| #26 | "Trochlear Fracture, Femur" |
| #27 | "Femoral Trochlear Fractures" |
| #28 | "Femoral Trochlear Fracture" |
| #29 | "Fracture, Femoral Trochlear" |
| #30 | "Fractures, Femoral Trochlear" |
| #31 | "Trochlear Fracture, Femoral" |
| #32 | "Trochlear Fractures, Femoral" |
| #33 | "Subtrochanteric Fractures" |
| #34 | "Subtrochanteric Fractures" |
| #35 | #15 OR #16 OR #17 OR #18 OR #19 OR #20 OR #21 OR #22 OR #23 OR #24 OR #25 OR #26 OR #27 OR #28 OR #29 OR #30 OR #31 OR #32 OR #33 OR #34 |
| #36 | #14 AND #35 |
| Filters | Results by year: from 1940 to 2024 (search date: 3 July 2024) |
|  | Language: English |
|  | Source: PUBMED |

**B. Search strategy of Embase through Embase**

| #1 | "Telerehabilitation" |
| --- | --- |
| #2 | "Telerehabilitations" |
| #3 | "Tele-rehabilitation" |
| #4 | "Tele rehabilitation" |
| #5 | "Tele-rehabilitations" |
| #6 | "Remote Rehabilitation" |
| #7 | "Rehabilitation, Remote" |
| #8 | "Rehabilitations, Remote" |
| #9 | "Remote Rehabilitations" |
| #10 | "Virtual Rehabilitation" |
| #11 | "Rehabilitation, Virtual" |
| #12 | "Rehabilitations, Virtual" |
| #13 | "Virtual Rehabilitations" |
| #14 | #1 OR #2 OR #3 OR #4 OR #5 OR #6 OR #7 OR #8 OR #9 OR #10 OR #11 OR #12 OR #13 |
| #15 | "Hip Fractures" |
| #16 | "Fractures, Hip" |
| #17 | "Intertrochanteric Fractures" |
| #18 | "Intertrochanteric Fractures" |
| #19 | "Trochanteric Fractures" |
| #20 | "Fractures, Trochanteric" |
| #21 | "Trochlear Fractures, Femur" |
| #22 | "Femur Trochlear Fracture" |
| #23 | "Femur Trochlear Fractures" |
| #24 | "Femur Trochlear Fractures" |
| #25 | "Fractures, Femur Trochlear" |
| #26 | "Trochlear Fracture, Femur" |
| #27 | "Femoral Trochlear Fractures" |
| #28 | "Femoral Trochlear Fracture" |
| #29 | "Fracture, Femoral Trochlear" |
| #30 | "Fractures, Femoral Trochlear" |
| #31 | "Trochlear Fracture, Femoral" |
| #32 | "Trochlear Fractures, Femoral" |
| #33 | "Subtrochanteric Fractures" |
| #34 | "Subtrochanteric Fractures" |
| #35 | #15 OR #16 OR #17 OR #18 OR #19 OR #20 OR #21 OR #22 OR #23 OR #24 OR #25 OR #26 OR #27 OR #28 OR #29 OR #30 OR #31 OR #32 OR #33 OR #34 |
| #36 | #14 AND #35 |
| Filters | Results by year: from 1940 to 2024 (search date: 3 July 2024) |
|  | Language: English |
|  | Source: EMBASE |

**C. Search strategy of CENTRAL through Cochrane Library**

| #1 | "Telerehabilitation" |
| --- | --- |
| #2 | "Telerehabilitations" |
| #3 | "Tele-rehabilitation" |
| #4 | "Tele rehabilitation" |
| #5 | "Tele-rehabilitations" |
| #6 | "Remote Rehabilitation" |
| #7 | "Rehabilitation, Remote" |
| #8 | "Rehabilitations, Remote" |
| #9 | "Remote Rehabilitations" |
| #10 | "Virtual Rehabilitation" |
| #11 | "Rehabilitation, Virtual" |
| #12 | "Rehabilitations, Virtual" |
| #13 | "Virtual Rehabilitations" |
| #14 | #1 OR #2 OR #3 OR #4 OR #5 OR #6 OR #7 OR #8 OR #9 OR #10 OR #11 OR #12 OR #13 |
| #15 | "Hip Fractures" |
| #16 | "Fractures, Hip" |
| #17 | "Intertrochanteric Fractures" |
| #18 | "Intertrochanteric Fractures" |
| #19 | "Trochanteric Fractures" |
| #20 | "Fractures, Trochanteric" |
| #21 | "Trochlear Fractures, Femur" |
| #22 | "Femur Trochlear Fracture" |
| #23 | "Femur Trochlear Fractures" |
| #24 | "Femur Trochlear Fractures" |
| #25 | "Fractures, Femur Trochlear" |
| #26 | "Trochlear Fracture, Femur" |
| #27 | "Femoral Trochlear Fractures" |
| #28 | "Femoral Trochlear Fracture" |
| #29 | "Fracture, Femoral Trochlear" |
| #30 | "Fractures, Femoral Trochlear" |
| #31 | "Trochlear Fracture, Femoral" |
| #32 | "Trochlear Fractures, Femoral" |
| #33 | "Subtrochanteric Fractures" |
| #34 | "Subtrochanteric Fractures" |
| #35 | #15 OR #16 OR #17 OR #18 OR #19 OR #20 OR #21 OR #22 OR #23 OR #24 OR #25 OR #26 OR #27 OR #28 OR #29 OR #30 OR #31 OR #32 OR #33 OR #34 |
| #36 | #14 AND #35 |
| Filters | Results by year: from 1940 to 2024 (search date: 3 July 2024) |
|  | Language: English |
|  | Source: Cochrane Library |

**D. Search strategy of WOB through Web of Science**

| #1 | "Telerehabilitation" |
| --- | --- |
| #2 | "Telerehabilitations" |
| #3 | "Tele-rehabilitation" |
| #4 | "Tele rehabilitation" |
| #5 | "Tele-rehabilitations" |
| #6 | "Remote Rehabilitation" |
| #7 | "Rehabilitation, Remote" |
| #8 | "Rehabilitations, Remote" |
| #9 | "Remote Rehabilitations" |
| #10 | "Virtual Rehabilitation" |
| #11 | "Rehabilitation, Virtual" |
| #12 | "Rehabilitations, Virtual" |
| #13 | "Virtual Rehabilitations" |
| #14 | #1 OR #2 OR #3 OR #4 OR #5 OR #6 OR #7 OR #8 OR #9 OR #10 OR #11 OR #12 OR #13 |
| #15 | "Hip Fractures" |
| #16 | "Fractures, Hip" |
| #17 | "Intertrochanteric Fractures" |
| #18 | "Intertrochanteric Fractures" |
| #19 | "Trochanteric Fractures" |
| #20 | "Fractures, Trochanteric" |
| #21 | "Trochlear Fractures, Femur" |
| #22 | "Femur Trochlear Fracture" |
| #23 | "Femur Trochlear Fractures" |
| #24 | "Femur Trochlear Fractures" |
| #25 | "Fractures, Femur Trochlear" |
| #26 | "Trochlear Fracture, Femur" |
| #27 | "Femoral Trochlear Fractures" |
| #28 | "Femoral Trochlear Fracture" |
| #29 | "Fracture, Femoral Trochlear" |
| #30 | "Fractures, Femoral Trochlear" |
| #31 | "Trochlear Fracture, Femoral" |
| #32 | "Trochlear Fractures, Femoral" |
| #33 | "Subtrochanteric Fractures" |
| #34 | "Subtrochanteric Fractures" |
| #35 | #15 OR #16 OR #17 OR #18 OR #19 OR #20 OR #21 OR #22 OR #23 OR #24 OR #25 OR #26 OR #27 OR #28 OR #29 OR #30 OR #31 OR #32 OR #33 OR #34 |
| #36 | #14 AND #35 |
| Filters | Results by year: from 1940 to 2024 (search date: 3 July 2024) |
|  | Language: English |
|  | Source: Web of Science |
